# Risk factors for outcome after allogeneic stem cell transplantation in patients with advanced phase CML

**DOI:** 10.1038/s41409-021-01410-x

**Published:** 2021-07-30

**Authors:** Christian Niederwieser, Elena Morozova, Ludmila Zubarovskaya, Tatjana Zabelina, Evgeny Klyuchnikov, Dietlinde Janson, Christine Wolschke, Maximilian Christopeit, Francis Ayuk, Ivan Moiseev, Boris V. Afanasyev, Nicolaus Kröger

**Affiliations:** 1 University Medical Center Hamburg Eppendorf, Department of Stem Cell Transplantation, Hamburg, Germany; 2 Raisa Gorbacheva Memorial Institute for Children Hematology and Transplantology, Saint Petersburg, Russian Federation

**Keywords:** Risk factors, Haematopoietic stem cells, Chronic myeloid leukaemia, Biostatistics, Bone marrow transplantation

## Abstract

Allogeneic hematopoietic stem-cell transplantation (HSCT) remains the only curative option for patients with advanced chronic myeloid leukemia (CML). However, outcome is dismal and of short follow-up. The objective of the study was to determine long-term outcome and risk factors in patients with a history of CML Blast Crisis (BC; *n* = 96) or accelerated phase (*n* = 51) transplanted between 1990 and 2018. At transplant, patients had a median age of 39 (range 7–76) years and were in ≥CP2 (*n* = 70), in AP (*n* = 40) or in BC (*n* = 37) with a diagnosis-HSCT interval of median 1.9 (range 0.3–24.4) years. Overall survival (OS) amounted 34% (95% CI 22–46) and progression-free survival (PFS) 26% (95% CI 16-36) at 15 years. Adverse risk factors for OS and PFS were low CD34^+^ count in the graft, donor age (>36 years) and BC. Cumulative incidence of Non-Relapse Mortality (NRM) was 28% (95% CI 18–38) and of relapse (RI) 43% (95% CI 33–53) at 15 years. PB-HSCT and HSCT after 2008 were favorable prognostic factors for NRM, while family donor and patient age >39 years were independently associated with higher RI. HSCT resulted in long-term OS in patients with advanced CML. OS was improved in non-BC patients, with donors ≤36 years and with higher CD34^+^ dose in the graft.

## Introduction

The introduction of tyrosine kinase inhibitors (TKI) for BCR/ABL in patients with chronic phase (CP) chronic myeloid leukemia (CML) resulted for the first time in regular disappearance of the malignant clone, restoration of normal life expectancy, and even treatment free remission. In contrast, the outcome of patients with TKI resistance or of those who present with advanced disease is still dismal. In such cases, the median overall survival (OS) with first and further TKI generations is less than 12 months [1–5]. Patients presenting with de novo blast crisis (BC), a distinct entity as compared to the accelerated phase (AP) and BC developing during TKI treatment, had remarkably adverse results [6]. Despite promising short term results with Ponatinib in subgroup of patients with resistance to TKI or T315I mutations [7], the use of second and third generation TKI has done little to change the overall outcome of BC and AP. Independent prognostic factors for increased risk of death in the TKI era were myeloid immunophenotype, prior TKI treatment, patient age ≥58 years, LDH ≥ 1227 IU/L, platelet count <102 GPT/l, no allogeneic stem cell transplantation (HSCT), blast phase from CP/AP and presence of chromosome 15 aberrations in patients with BC [1].

HSCT remains the only curative option in advanced phase, but data on outcome are scarce. Results in BC are clearly inferior to those of CP1 pointing to an unmet need for improvement [8]. For patients in CP after BC at HSCT, age ≥45 years, lower performance status (<80%), longer interval from BC diagnosis to HSCT (>12 months), myeloablative conditioning and unrelated HSCT were risk factors for inferior survival in a multicenter (*n* = 46) study [9].

The purpose of the study was to evaluate long-term results (over 15 years) on a large number of patients with advanced CML and analyze risk factors for outcome. Therefore, we analyzed outcome and risk factors in 147 patients with advanced disease (BC, AP and CP ≥ 2) transplanted in two centers with a follow-up period of up to 15 years.

## Patients and methods

All patients with advanced disease (*n* = 147) transplanted between 1990 and October 2018 at the University Hospital of Hamburg Eppendorf, Germany and the Raisa Gorbacheva Memorial Institute for Children Hematology and Transplantology (RGMI), Saint Petersburg, Russian Federation were analyzed (Table 1). Clinical, cytogenetic, and molecular characteristics of CML including mutational status and additional gene mutations at diagnosis and at HSCT are provided in Table 1. HSCT was performed according to standard protocols approved by the Ethical Committee of the University Medical Center Hamburg, Eppendorf, Germany and The Raisa Gorbacheva Memorial Institute, RGMI, St. Petersburg, Russian Federation, and after written informed consent including consent for data collection and analysis. TKI was given to 40 patients because of MRD^+^ (defined as BCR-ABL1/ ABL1 transcript ratio of <0.1% on two consecutive measurements) or prophylaxis, nine for hematological relapse, while 96 received no TKI post-HCT. Follow-up was performed at given intervals. Assessment of *BCR-ABL/ABL* transcript levels was done by quantitative real time (RT)-PCR at the departments laboratories according to the updated guidelines of the European LeukemiaNet (ELN) [10]. Pre-established donor/recipient-specific polymorphism were used in all patients post-HSCT for donor chimerism studies. Y-chromosome-specific sequences were determined in sex-mismatched transplants by validated molecular quantitative RT-PCR techniques, which guarantee high sensitivity [10^–4^] for the assessment of chimerism [11, 12].

**Table 1:** Patients characteristics.

	Time point					Donor
	at diagnosis	diagnosis- HSCT	at HSCT		after HSCT	
Variable	*n* (%)					
*n* (UKE Hamburg/Saint Petersburg)	147 (79/68)					147
Age; median (range) years	39 (7–76)					36 [(14–66)
Children/Adults	8 (5.4)/139 (94.6)					1 (0.7)/146
Gender male	94 (63.9)					100 (68.0)
Disease stage						
Blast Crisis	17 (11.6)	43 (29.3)		37 (25.2)		
at any time	96 (65.3)					
Age ≤39 />39 years				16/21		
non Blast Crisis	130 (88.4)	104 (70.7)		110 (74.8)		
at any time	51 (34.7)					
Age ≤39 />39 years				61/49		
Cytogenetics (*n* = 76) Ph^+^ only	(75)	n.d.				
Ph^+^ and complex	(12)	n.d.				
Ph^+^ +8	(5)	n.d.				
Ph^+^ and (del[9], del[17], *t*(3;2), 2xt(3;21), *t*(2;19)	(8)	n.d.				
Molecular (*n* = 43) bcr/abl	(100)	n.d.				
no mutations	(88.4)	n.d.				
T315I mutation	(7)	n.d.				
E499E, F359C, H396R, S317L, F317L, V299L, Y253H	(11.6)	n.d.				
Additional mutations (TET2, DNMT3A, SF3B1)	(2.3)	n.d.				
Tyrosine kinase inhibitors (TKI) Yes	119 (81.5)				49 (33.8)^a^	
No	27 (18.5)				96 (66.2)	
Generation of TKI 1st	40 (27.2)				8 (10)	
1st + 2nd/1st + 3nd	20 (13.6)/1 (0.7)				0	
2nd ± 3n/3rd	57 (38.1) / 1 (0.7)				11 (14)/0	
CMV status (IgG) positive	100 (68.0)					84 (57.1)
negative	47 (32.0)					63 (42.9)
Diagnosis - HSCT interval; median (range) years	1.9 (0.3–24.4)					
Conditioning, age, and disease stage						
Standard myeloablative (MAC)				63 (42.9)		
patient age (≤39/>39)				32/31		
Blast Crisis/non-Blast Crisis				14/49		
Reduced intensity (RIC)				84 (57.1)		
patient age (≤39/>39)				45/39		
Blast Crisis/non-Blast Crisis				23/61		
ATG as conditioning				77 (52.4)		
HLA compatibility Matched/mismatched RD				47 (32.0)/6 (4.0)		
Matched/mismatched UD				58 (39.5)/36 (24.5)		
Stem cell source PBSC/BM				85 (57.8)/62 (42.2)		
CD 34^+^ count; median (range) × 10^6^/kg bw				5.4 (0.4–19)		

*HSCT* hematopoetic stem cell transplantation, *BC* blast crisis, *CP* chronic phase, *ATG* antithymocyte globulin, *HLA* human leukocyte antigens, *n.d.* not determined, *bw* body weight, *RD* related donor, *UR* unrelated donor, *PBSC* peripheral blood stem cells, *BM* bone marrow, MAC: Busulfan/cyclophosphamide (*n* = 40), Flamsa (*n* = 15), TBI/cyclophosphamide (*n* = 8); RIC: Fludarabin/Buslufan or Melphalan (*n* = 81), Flamsa (*n* = 1), Cyclophosphamide/TT (*n* = 2).

^a^Nine patients had TKI for treatment of relapse.

### Study endpoints and definitions

Primary endpoints of this retrospective analysis were long-term overall survival (OS) calculated from the date of HSCT to death due to any cause. Secondary endpoints were progression free survival (PFS), incidence of NRM (date of HSCT to date of death in the absence of disease relapse) and RI (from date of HSCT to date of relapse). For all endpoints, patients alive were censored at the date of last contact. ELN criteria were applied for the definition of BC, AP, remission, and relapse [13]. Acute and chronic GvHD were graded and reported according to the standard clinical criteria. The first of three consecutive days with white blood cell count (WBC) > 1.0 gpt/L was considered as leucocyte engraftment and >20.000/µL platelets without transfusion as platelet engraftment. Primary graft failure was defined as no engraftment within 28 days and relapse according to reappearance of molecular, cytogenetic, and hematological disease characteristics as molecular, cytogenetic, and hematological relapse.

### Statistics

Characteristics of patients were expressed as median and range for continuous variables and frequencies for categorical variables. Categorical data were compared by chi-square test or Fisher’s exact test. The probability of OS and PFS was calculated using the Kaplan-Meier estimator. The log-rank test was used to compare survival curves. All variables with *p* ≤ 0.1 were entered in a multivariable Cox regression model (backward elimination using the Wald test). Only results of the final models are presented as relative risks (hazard ratios [HRs]) with respect to a reference category (HR, 1) together with the 95% confidence interval and *p* values. The cumulative incidence method was used to estimate the incidence of NRM and relapse to account for competing events. The Gray test was used to compare cumulative incidence curves. Calculations were performed with SPSS, version 22 (SPSS, Chicago, IL), and the competing risk analyses was performed in ACCorD statistics software (V. Gebski, NHMRC Clinical Trials Centre, University of Sydney).

## Results

Patient characteristics are given in Table 1 including patient age [median 39 (7–76) years] and donor age [36 (14–66) years], gender and CMV positivity. Patients had de novo BC in 11.6%, BC at any time before HSCT in 65.3% and at HSCT 25.2%. At the time of HSCT patients were in CP ≥ 2 (*n* = 70), AP (*n* = 40), or BC (*n* = 37). The majority were treated with TKI (81.5%) before HSCT and the median diagnosis-HSCT interval amounted to 1.9 (range 0.3–24.4) years. Reduced intensity conditioning was given to 57.1% of the patients because of age and/or comorbidities. Of the 45 younger patients with RIC, 84% had non-BC at HSCT, had intensive pretreatment and comorbidities making them ineligible for MAC. Transplant characteristics, graft source, GvHD prophylaxis and post transplant TKI treatment is given in Table 1. Post-HSCT TKI was given for MRD^+^ or relapse prophylaxis to 40, for treatment of relapse to 9 and 96 patients did not receive TKIs.

**Fig. 1:**
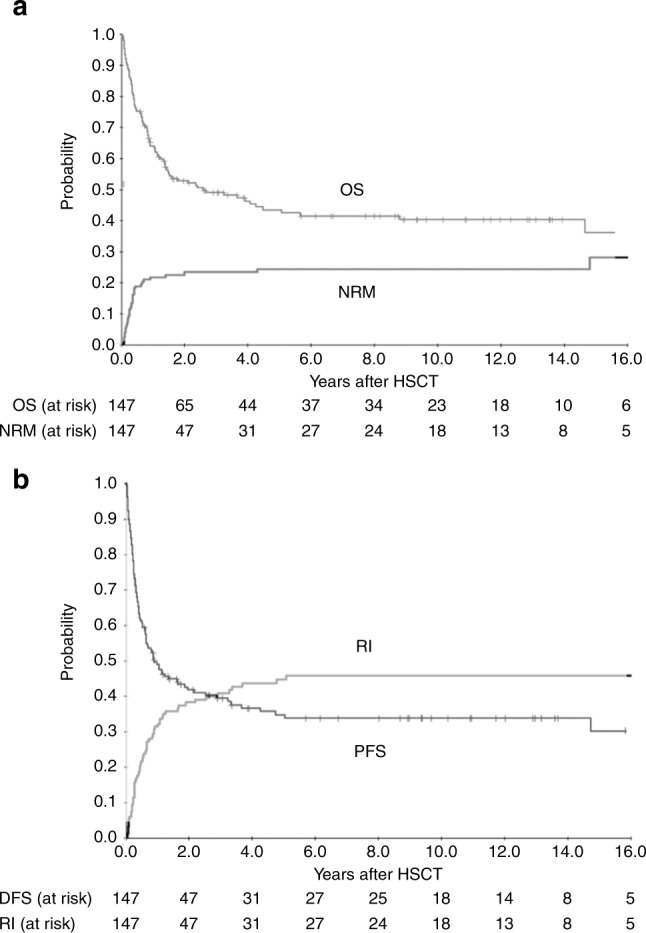
Outcome of patients with advanced CML (*n* = 147) after HSCT. **a** Overall Survival (OS) and cumulative incidence of Non-Relapse Mortality (NRM) **b** Progression Free Survival (PFS) and cumulative Relapse Incidence (RI).

**Fig. 2:**
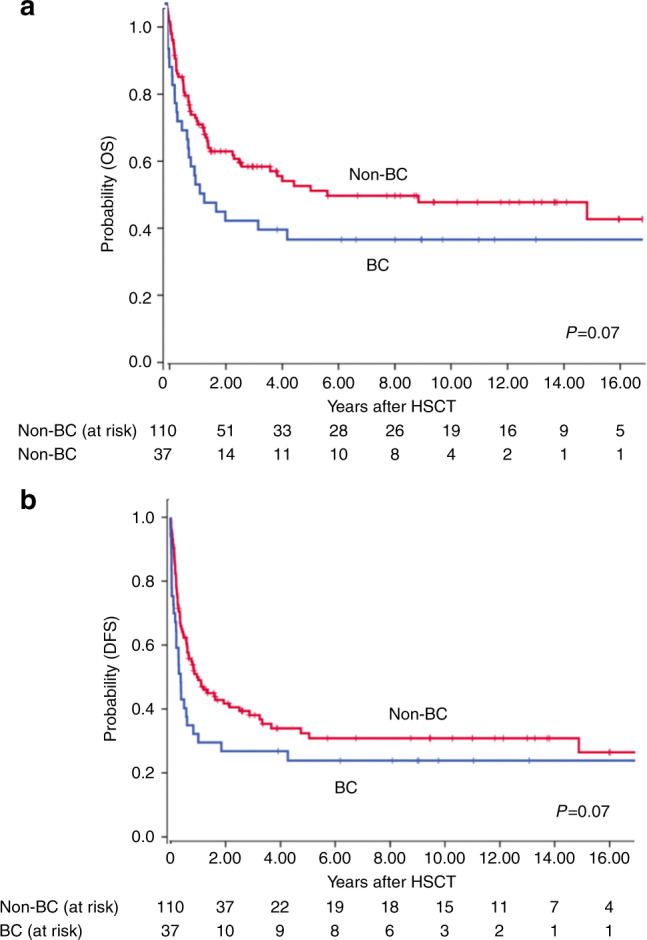
Outcome of patients according to disease stage (BC and non-BC) after HSCT (*n* = 147). **a** Overall survival (OS) **b** Progression free survival (PFS).

Of the 147 patients, 93.9% engrafted, one progressed immediately after HSCT and 5.4% had primary graft failure (Table 2). WBC engraftment was observed a median of 16 (range 9–39) and platelet engraftment a median of 17 (range 6–63) days post-HSCT. With a median follow-up of 9 years, OS of all patients amounted to 38% (95% CI 30–46) at 10 years and 34% (95% CI 22–46) at 15 years, while PFS reached 30% (95% CI 22–38) at 10 years and 26% (95% CI 16–36) at 15 years (Fig. 1). History of BC before HSCT did not influence OS, but phase at HSCT. Patients with BC independent of myeloid or lymphoid origin at HSCT had a trend for worse OS as compared to AP or ≥CP2 (Fig. 2; *p* = 0.07, 41% vs 30% at 10 years) but OS in patients with AP at transplantation was not significantly different compared to those with ≥CP2. Interestingly, no difference was observed in patients with de novo BC as compared to the rest (Figure S1).

**Table 2:** Results.

Variable	*n* (%)
Follow-up years; median (range)	9 (3.4–13.2)
Engraftment; *n* (%)	
yes	138 (93.9)
Primary graft failure	8 (5.4)
Progression before engraftment	1 (0.7)
Hematopoietic reconstitution; median (range) days	
WBC	16 [9–39]
platelets	17 [6–63]
Acute GvHD, *n* (%)	
0–I	84 (61.3)
II–IV	53 (38.7)
Cumulative Incidence acute GvHD II-IV at day 100	34% (95% CI 26–42)
Chronic GvHD; *n* (%)	45 (38.0)
none	75 (62.5)
mild	15 (12.5)
moderate	25 (20.8)
severe	5 (4.2)
Cumulative incidence chronic GvHD at 1 year	24% (95% CI 16–32)
Relapse:	64
Molecular	18 (28.1)
Cytogenetic	4 (6.3)
Hematological	42 (65.6)
Relapse on TKI (prophylaxis and MRD^+^)	17 (42.5)
Relapse on no TKI	40 (38.1)
Treamtent of relapse with DLI	34
Cause of death; *n* (%)	85 (57.8)
Relapse/progress of primary disease	49 (57.6)
GvHD (acute and chronic)	15 (17.7)
Infection	11 (12.9)
Primary graft failure	5 (5.9)
Secondary graft failure, infectious	2 (2.4)
Secondary malignancy	2 (2.4)
VOD	1 (1.2)

*WBC* white blood cells, *DLI* Donor lymphocyte infusion, *GvHD* graft-versus-host disease, *VOD* veno-occlusive-disease.

NRM amounted 1% (95% CI 0–2) and 12% (95% CI 6–18) on day 30 and 100, respectively. Cumulative incidence of NRM amounted to 25% (95% CI 17–33) at 5 years and 28% (95% CI 18–38) at 15 years (Fig. 1a). The causes of death for NRM were GvHD (17.7%), infections (12.9%), primary and secondary graft failure (8.3%), secondary malignancies (2.4%), and VOD (1.2%). Except for a patient with secondary malignancy, there was a plateau after 2 years for NRM.

The median time for molecular relapse was 5.3 (range 0.7–44.4) months. A total of 57 patients had hematological relapse, 17 despite receiving TKI and 40 without TKI (Table 2). TKI treatment positively influenced OS (*p* < 0.001; Figure S2). Hematological RI amounted to 43% (95% CI 33–53) at 15 years, median time to hematological relapse was 10.3 (range 1–61) months and plateauing after 5 years. Of the two groups with molecular or cytogenetic relapse the mortality rate was 50.0%, whereas for those with hematological relapse the mortality rate was 92.9%. DLI were given in 34 patients (Table 2). Patients receiving DLIs had a better OS than patients without DLI (Figure S3; *p* < 0.001). The most frequent causes of death was relapse of the primary disease (57.6%). Fatality rate at relapse was related to the status at HSCT. A total of 32 (71.1%) out of the 45 patients with non-BC and 17 (89.5%) out of 19 patients with BC at HSCT died.

### Prognostic factors

Patient, donor, and transplant characteristics were analyzed for associations with outcome in univariate analyses at 5 years (Table 3). Lower CD34^+^ cell count ×10^6^/kg body weight (bw) in the graft associated with shorter OS [Hazard ratio (HR) 1.18 95% CI 1.0–1.37; *p* < 0.01], as did BM 30% (95% CI 18–42) versus PBSC 46% (95% CI 35–58; *p* = 0.01). However, when CD34^+^ count was analyzed separately in patients with BM-HSCT and PB-HSCT, only patients with BM and not PB grafts showed a significant association between CD34^+^ count and OS (HR 1.18 95% CI 1.01–1.37; *p* = 0.04). Donor age >36 years was also identified as a risk factor for OS [33% (95% CI 21–45) versus 48% (95% CI 36–60) for donors >36 years and ≤36, respectively; *p* = 0.05] as was Karnofsky index <90% and ≥90% influenced OS (*p* = 0.002) and DFS (*p* = 0.002). Likewise, BC at HSCT associated negatively with OS with a trend [30% (95% CI 14–46) versus 44% (95% CI 34–54) for patients with BC and non-BC, respectively; *p* = 0.07]. MAC versus RIC did not influence OS and PFS (*p* = 0.89 and *p* = 0.611, respectively). The influence of CD34^+^ dose, stem cell source and a trend in donor age was confirmed in analysis of adult patients only (*p* = 0.02, *p* = 0.03, and *p* = 0.09, respectively).

**Table 3:** Univariate analyses of risk factors for overall survival (OS), progression free survival (PFS), cumulative incidence of non-relapse mortality (NRM), and cumulative relapse incidence (RI).

Factors	OS at 5 y			PFS at 5 y			NRM at 5 y			RI at 5 y		
	% (95% CI)	events	*p* value	% (95% CI)	events	*p* value	% (95% CI)	events	*p* value	% (95% CI)	events	*p* value
CD34^+^ count continuous	HR 1.18		<0.01	HR 1.10		<0.01						
	(95% CI 1.0–1.37)			(95% CI 1.03–1.18)								
Stem cell source:			0.01			<0.01			<0.01			0.78
Bone marrow	30 [18–42]	42		17 [7–27]	49		38 [26–50]	23		45 [31–59]	26	
PBSC	46 [34–58]	43		39 [27–51]	50		15 [7–23]	13		46 [34–58]	37	
Donor age:			0.05			0.09			0.89			0.25
≤36 years	48 [36–60]	38		35 [23–47]	47		25 [15–35]	18		40 [28–52]	29	
>36 years	33 [21–45]	46		24 [12–36]	51		24 [14–34]	18		51 [39–63]	33	
Disease status at HSCT:			0.07			0.07			0.99			0.17
Non-BC	44 [34–54]	59		31 [21–41]	71		24 [16–32]	27		45 [35–55]	44	
BC	30 [14–46]	26		24 [10–38]	28		24 [10–38]	9		51 [35–67]	19	
Patient gender:			0.2			0.06			0.18			0.48
Male	35 [25–45]	57		23 [13–33]	68		29 [19–39]	26		49 [37–61]	42	
Female	47 [33–61]	28		41 [27–55]	31		17 [7–27]	10		42 [28–56]	21	
Patients age:			0.35			0.61			0.05			0.03
≤39 years	36 [24–48]	47		29 [17–41]	51		32 [20–44]	24		38 [26–50]	27	
>39 years	45 [33–57]	38		30 [18–42]	48		16 [6–26]	12		54 [42–66]	36	
HSCT year 2008			0.24			0.78			0.02			0.1
before	36 [24–48]	49		27 [17–37]	54		33 [21–45]	25		40 [28–52]	29	
after	45 [31–59]	36		31 [17–45]	45		15 [7–23]	11		53 [39–67]	34	
Donor			0.3			0.16			0.46			0.02
Related	42 [30–54]	34		25 [13–37]	39		17 [7–27]	10		58 [44–72]	29	
Unrelated	34 [20–48]	51		32 [22–42]	60		29 [19–39]	26		39 [29–49]	34	
CD34^+^ ×10^6^/kg bw			0.01			0.02			0.84			0.04
≤5.4	31 [19–43]	46		17 [5–29]	53		23 [13–33]	16		60 [46–74]	37	
>5.4	50 [38–62]	34		41 [29–53]	41		22 [12–22]	16		37 [25–49]	25	
CMV IgG patient/donor			0.28			0.48			0.02			0.21
patient pos/donor neg	30 [10–50]^a^	23		27 [11–43]^a^	26		43 [33–53]^a^	14		37 [19–55]^a^	12	
all others	42 [32–52]	62		32 [22–42]	73		19 [11–27]	22		49 [39–59]	51	

*OS* overall survival, *BC* blast crisis, *BW* body weight, *HR* hazard ratio, *PFS* progression-free survival, *NRM* non-relapse mortality, *RI* relapse incidence, *CI* Confidence interval, *PBSC* peripheral blood stem cells, *pos* positive, *neg* negative

^a^Shorter follow-up period

In multivariable analyses for OS on the whole population, donor age >36 years (HR 1.74 95% CI 1.11–2.71; *p* = 0.02), BC at HSCT (HR 1.85 95% CI 1.13–3.04; *p* = 0.01) and lower CD34^+^ cell dose (HR 1.12 95% CI 1.04–1.20; *p* = 0.003 and HR 2.14 95% CI 1.33–3.45 using categorical variables) were independently associated with shorter OS (Table 4).

**Table 4:** Multivariate Cox regression analysis of risk factors for overall survival (OS), progression-free survival (PFS), non-relapse mortality (NRM), and relapse incidence (RI).

Variable^a^	OS			PFS			NRM			RI		
	HR (95% CI)	Events ref/var	*p*	HR (95% CI)	Events ref/var	*p*	HR (95% CI)	events	*p*	HR (95% CI)	events	*p*
Donor age (>36 years)	1.74 (1.11–2.71)	75/65	0.02	1.62 (1.07–2.44)	75/65	0.02						
Blast crisis at HSCT	1.85 (1.13–3.04)	105/35	0.01	1.76 (1.11–2.8)	35/105	0.02				1.78 (0.98–3.21)^b^	141	0.06
CD34^+^ (Continuous/*categorical*)	1.12 (1.04–1.20)/2.14 (1.33–3.45)^c^	70/70	<0.01/<0.01^c^	1.12 (1.05–1.20)/1.67 (1.06–2.66)^c^	70/70	<0.01/<0.03^c^				1.61 (0.95–2.78)^b^	*141*	*0.08*
Stem cell source PBSC							0.34 (0.18–0.67)	140	<0.01			
HSCT after year 2008							0.40 (0.20–0.82)	140	0.01			
related donor										1.97 (1.18–3.29)	141	0.01
Patient age > 39 years										1.62 (0.98–2.69)	141	0.06

*HR* hazard ratio, *CI* confidence interval, *PBSC* peripheral blood stem cell

^a^All variables from the univariate analyses with *p* ≤ 0.1 were entered in a multivariable Cox regression model (backward elimination using the Wald test).

^b^Independent variables for RI using factors significant in univariate analyses for OS (statistical trend).

^c^Categorical values for CD34^+^ for OS and PFS are entered from an additional analysis.

Similarly, lower CD34^+^ cell count in the graft (HR 1.10 95% CI 1.03–1.18 *p* = 0.003) and BM (*p* = 0.005) were associated with worse PFS. In addition, donor age >36 years [24% (95% CI 12–36) versus 35% (95% CI 23–47) with younger donors; *p* = 0.09], BC at HSCT [24% (95% CI 10–38) versus 31% (95% CI 21–41) for non-BC; *p* = 0.07] and male patient gender were associated with a trend for worse PFS in univariate analyses [23% (95% CI 13–33) versus 41% (95% CI 27–55) for female patients; Table 3]. In multivariable analyses, donor age >36 years (HR 1.62 95% CI 1.07–2.44; *p* = 0.02), BC at HSCT (HR 1.76 95% CI 1.11–2.80; *p* = 0.02) and lower CD34^+^ dose (HR 1.12 95% CI 1.05–1.20; *p* = 0.001) for continous and for categorical (HR 1.67 95% CI 1.06–2.66) were independently associated with shorter PFS (Table 4).

There was a higher cumulative incidence of NRM at 5 years in patients receiving BM grafts [38% (95% CI 26–50) versus 15% (95% CI 7–23) in PB; *p* = 0.003] and in patients ≤39 years [32% (95% CI 20–44) versus 16% (95% CI 6–26) in older patients; *p* = 0.05]. HSCT before the year 2008 [33% (95% CI 21–45) versus 15% (95% CI 7–23) after 2008; *p* = 0.02] and a patient positive and donor negative CMV constellation [43% (95% CI 33–53) at 3 y versus 19% (95% CI 11–27) at 5 y; *p* = 0.02] was associated with higher NRM. Only stem cell source [PB-HSCT HR 0.34 95% CI 0.18–0.67; *p* < 0.01] and HSCT after 2008 [HR 0.40 95% CI 0.20–0.82; *p* = 0.01] were favorably associated with NRM in multivariable analyses. The evaluation of RIC versus MAC did not associate independently with NRM [HR 0.57 95% CI 0.30–1.10; *p* = 0.09].

**Fig. 3:**
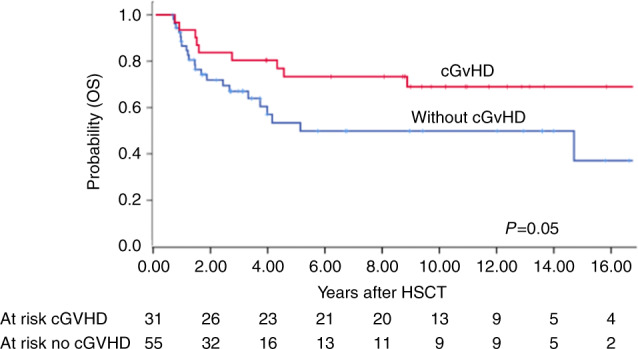
Landmark analysis in disease-free patients with advanced CML 180 days after HSCT. Overall survival according to presence and absence of chronic GvHD.

Cumulative Incidence of acute GvHD II–IV day 100 was 34% (95% CI 26–42) and cumulative incidence of chronic GvHD at 1 year was 24% (95% CI 16–32) (Table 2). In a landmark analysis in patients disease free after 180 days (*p* = 0.05) but not after 365 days chronic GvHD influenced OS (Fig. 3).

Higher cumulative RI (at 5 years) was observed in patients aged >39 years [54% (95% CI 42–66) versus 38 % (95% CI 26–50) in younger patients-; *p* = 0.03) and in patients with HSCT from related donors in comparison to unrelated donors [58% (95% CI 44–72) versus 39% (95% CI 29–49), respectively; *p* = 0.02; see Table 3). Furthermore, CD34^+^ count of ≤5.4 × 10^6^/kg bw in the graft [60% (95% CI 46–74) versus 37% (95% CI 25–49) in >5.4 × 10^6^/kg bw; *p* = 0.04) was associated with higher cumulative RI. A family donor (HR 1.97 95% CI 1.18–3.29; *p* = 0.01) remained associated with higher RI and patient age >39 years (HR 1.62 95% CI 0.98–2.69; *p* = 0.06) with a trend in multivariable analysis.

## Discussion

In this study, the long-term outcome of 147 patients after HSCT for advanced phase CML resulted in 34% at 15 years OS and 26% at 15 years PFS. OS was influenced by stage of advanced disease at HSCT and was 36% after ≥CP2, 45% after AP, and 30% after BP at 14 years. In addition, we found that low CD34^+^ cell count/kg bw in the graft, donor age >36 years, and BC were unfavorable independent prognostic markers for OS and PFS. BM and HSCT before 2008 were independently associated with higher NRM, and related donor and patients age (>39 years) with higher RI.

The current results extend our previous evaluation [8] on advanced phase CML with twice the patient numbers and an observation period extended to 15 years. Previously published results had OS of 43% with a plateau at 3 years, while here OS was 41% at 5 years, 38% at 10 years, and 34% at 15 years. Similarly, PFS of 32% at 2 years is now 31% at 5 years, 30% at 10 years, and 26% at 15 years. There were no relapses 5 years after HSCT and RI has plateaued since, confirming the curative potential of HSCT in advanced phase in both transplant centers (superimposable outcome; Figure S4; *p* = 0.55). Our results compared favorably with those previously published for advanced phase CML showing 23.8% OS at 3 years despite being transplanted earlier in the course of the disease [9] and are similar to the results published by the German CML study group [14]. In our analysis, OS of patients with active BC at the time of HSCT amounted to 35.1% at 3 years, while the OS previously reported for patients with advanced phase (but not only in BC) was 51.3% at 3 years. Interestingly, OS in de novo BC was not different from BC, AP and ≥CP2.

Our results compare also favorably with the one of patients with TKI resistance or T315I mutation treated with ponatinib alone. Despite having short follow up, BC patients treated with ponatinib alone had inferior results than in our cohort and patients with AP (*n* = 18) had similar short term results in both studies, but missing long-term results (follow up in the two studies is 36 months versus 16 years in our study). Future studies investigating the promising concept of sequential combination Ponatinib and HSCT are warranted.

CD34^+^ cell count/kg bw in the graft has been shown to be predictive for survival in different haematological diseases following PB-HSCT (CD34^+^ > 4.5 × 10^6^/kg bw only 18 CML patients) and BM-HSCT (CD34^+^ > 3 × 10^6^/kg bw; 55 patients with CML, of which 11 accelerated phase and 2 in BC) [15, 16]. CD34^+^ cell dose (>2.5 × 10^6^/kg bw) was found to influence OS in 99 AML patients transplanted with BM grafts from HLA identical siblings [17]. We found higher CD34^+^ dose to be associated with better OS and PFS. This correlation was especially significant in patients transplanted with BM. Related (RD) and unrelated donors (UD) may have influenced indirectly CD34^+^ cell dose following differences in donor age. While there was no difference in patients age [median 39 (7–76) years and 39 (20–71) years in UD and RD, respectively], donor age was lower in UD [median 39 (14–66) years and median 31 (20–58) years for related and unrelated donors, respectively (*p* < 0.001)]. Possible explanations for this effect may also involve the stage of the disease (CML in advanced phase) and the higher number of CD34^+^ (median 5.4 × 10^6^/kg bw) used in our cohort as compared to the previously published results. The multivariable analysis confirmed that the CD34^+^ dose and not the graft source influenced outcome. Furthermore, a multivariate analyses only on patients transplanted with BM grafts, which usually have less CD34^+^ cells, confirmed CD34^+^ cell dose as an independent variable (data not shown). Accellerated engraftment after higher CD34^+^ cell dose may decrease NRM and possibly lead to a lower RI (see univariable analysis) finally resulting in better outcome.

The advantage of PB-HSCT over BM-HSCT has been a matter of debate for several decades and is associated indirectly with higher CD34^+^ cell dose. PB-HSCT has been shown to have faster neutrophil and platelet engraftment and less severe acute and extensive chronic GvHD [18]. Another publication reported that PB-HSCT was not inferior to BM-HSCT in high-risk disease [19]. While BM is the preferred source for chronic phase, PB-HSCT has been described to be similar to BM for OS, relapse, and NRM in patients with advanced CML [20]. In our cohort, PB-HSCT was an independent beneficial factor for NRM, but not for OS or PFS.

Another independent and highly significant risk factor for OS and PFS in our extended study was advanced disease stage at HSCT (BC vs non-BC). It has been shown previously that BC at HSCT was associated with extremely poor prognosis [21, 22]. In our cohort, BC at HSCT was an essential risk factor for OS. Efforts to downgrade the disease to non-BC should therefore be undertaken with TKI and, if unsuccessful, with intensive chemotherapy [23, 24].

Higher donor age was a new important risk factor for outcome after HSCT in advanced CML and particularly important in HSCT from unrelated donors, where donors may be chosen by younger age. The results published to date are conflicting. Donor age >30 years was not prognostic for survival in patients with chronic phase CML after matched related HSCT [25]. Another analysis on patients with AML did not find donor age to be a risk factor for OS or DFS [26]. However, older donor age (≥30 years) has been associated with increased NRM in unrelated HSCT without influencing OS or LFS [27]. A further publication in standard-risk patients with heterogeneous diseases reported a beneficial effect of younger donors (<37 years) on OS and TRM [28, 29]. Our study, together with a publication in myelodysplastic syndrome [30], of patients with advanced-stage CML underscores the importance of donor age ≤ 36 years for OS and DFS. This may not apply to matched related donors >60 years, where the results were comparable to younger related donors [31, 32]. The increased presence of clonal hematopoiesis in healthy individuals >40 years of age [33] might influence outcome resulting in predisposition to malignant disease.

NRM was favorably influenced by PB and year of HSCT. Improvement of supportive therapy and high-resolution typing after 2008 might be responsible for this effect. Improvement over time (after 2006 and after 2010) has been reported earlier [9, 34].

Donor type and patient age were both important for RI. Family donors were associated with a higher incidence of relapse. Differences in minor histocompatibility antigens in unrelated HSCT leading to higher graft-versus-tumor effect may be responsible for this observation. Higher patient age may be associated with higher leukemic or non-leukemic mutations [35] and therefore might be associated with higher relapse incidence. In addition, a higher rate of reduced intensity conditioning in older patients might explain a higher RI. The high relapse incidence was caused by resistance to TKI and limited availability of further TKI generations. Optimised transcript assessment and prophylactic TKI treatment will contribute to lower RI in the future.

This analysis has beside several strengths (high number of patients including elderly; high number of comorbidities, bi-center study, and long term follow-up) but also limitations considering the retrospective nature of the evaluation and the inclusion of a considerable proportion of patients prior to the TKI era. In addition, we could not evaluate our results considering the EBMT CML score, which has been shown to influence survival predominantly in BM transplanted patients and before the TKI era [36]. The Haematopoietic HCT CI score (available only after 2005) could also not be considered [37].

In conclusion, this analysis of advanced phase CML supports the use of younger donors, the highest CD34^+^ cell dose (highest cell dose in our cohort 19 × 10^6^/kg bw) and the need to enter a non-BC phase before HSCT. In comparison to earlier publications, results in BC have improved considerably and may be further optimized by decreasing the current RI of 45% using more frequently maintenance TKI and/or MRD-tailored DLI.

## Supplementary information

CML Advanced phase supplemental material
