# Supplementary material for: Risk factors for outcome after allogeneic stem cell transplantation in patients with advanced phase CML
Source: Bone Marrow Transplant. 2021 Jul 30;56(11):2834–41. doi: 10.1038/s41409-021-01410-x (PMC8563424; doi:10.1038/s41409-021-01410-x)
Supplement: Supplementary file 1 — CML Advanced phase supplemental material [file 41409_2021_1410_MOESM1_ESM.docx]

Supplemental material:

Figure S1:

Comparison of OS of de novo BC versus patients in CP≥2 or accelerated phase (CPAP) at diagnosis

Figure S2:

OS of patients with (relapse, MRD+ or prophylactic n=49) or without TKI (n=96)

Figure S3:

DLI after relapse (n=34) and no DLI after relapse (n=25).

Figure S4:

OS in patients transplanted in Saint Petersburg (RGMI; n=68) and UKE Hamburg (n=79)

Figure S1

**
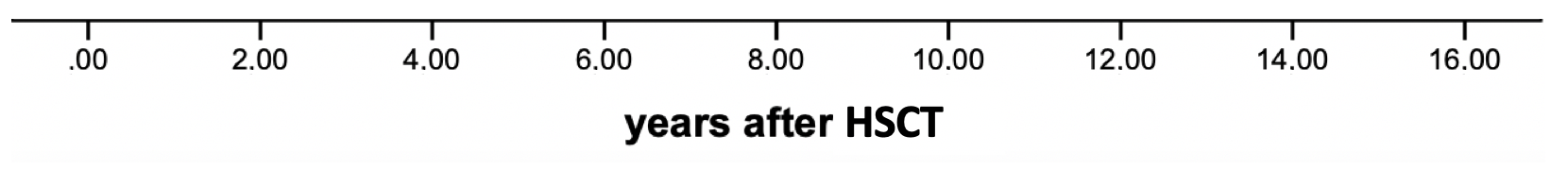
**


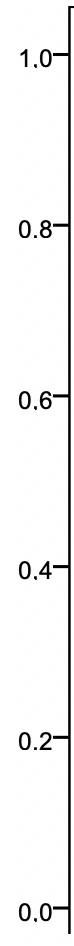


p=0.42

**probability (OS)**

| CPAP at diagnosis (at risk) | 130 | 58 | 43 | 37 | 33 | 22 | 17 | 9 | 5 |
| --- | --- | --- | --- | --- | --- | --- | --- | --- | --- |
| BC de novo (at risk) | 17 | 7 | 1 | 1 | 1 | 1 | 1 | 1 | 1 |

Figure S2

| TKI after HSCT (at risk) | 49 | 28 | 14 | 12 | 9 | 4 | 4 | 2 | 1 |
| --- | --- | --- | --- | --- | --- | --- | --- | --- | --- |
| No TKI after SCT (at risk) | 96 | 36 | 29 | 25 | 24 | 19 | 14 | 8 | 5 |

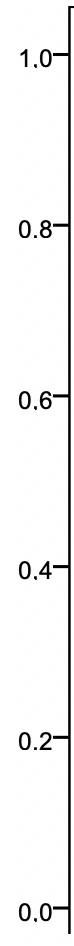


**
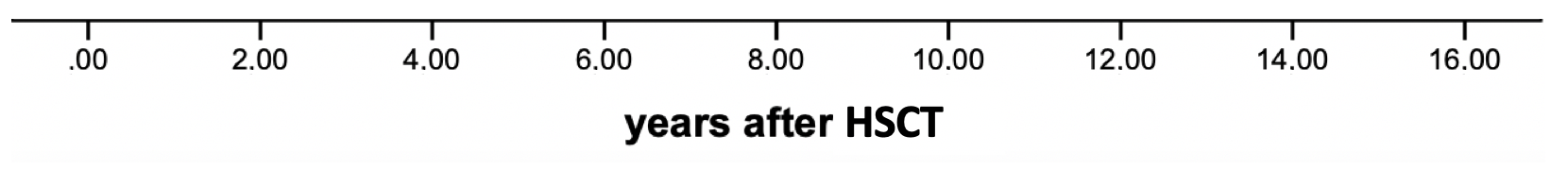
**

P<0.001

**probability (OS)**

Figure S3


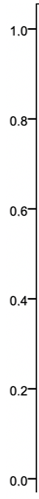


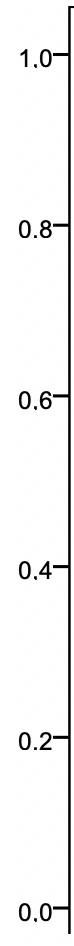


**probability (OS)**

p<0.001

| DLI after relapse (at risk) | 34 | 20 | 12 | 10 | 8 | 4 | 4 | 2 | 1 |
| --- | --- | --- | --- | --- | --- | --- | --- | --- | --- |
| no DLI after relapse (at risk) | 25 | 5 | 3 | 1 | 1 | 1 | 0 | 0 | 0 |

**
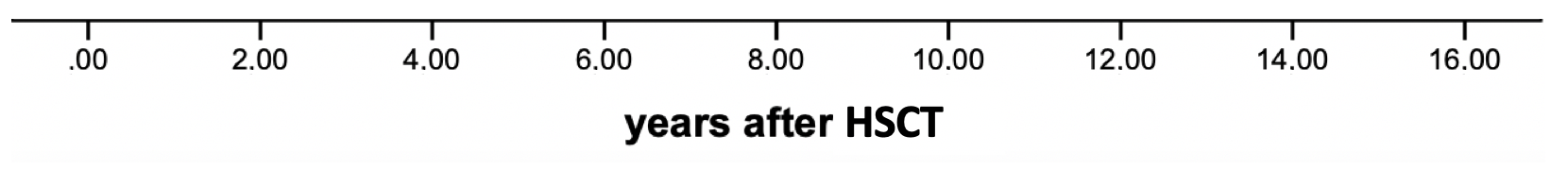
**

| days | | | 0 | | 100 | | 200 | | 300 | | 400 | | 500 | | 600 | | 700 |
| --- | --- | --- | --- | --- | --- | --- | --- | --- | --- | --- | --- | --- | --- | --- | --- | --- | --- |
| cGVHD (at risk) | 147 |  | |  | |  | |  | |  | |  | |  | |  |  |

Figure S4

p=n.s.


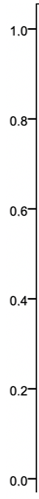


**
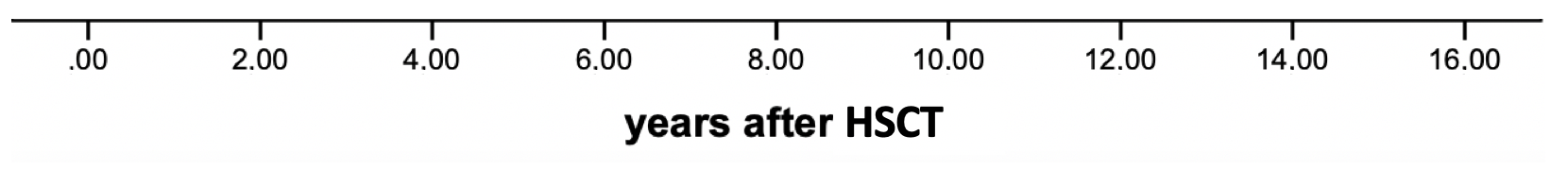
**

**probability (OS)**

| UKE Hamburg | 79 | 40 | 33 | 30 | 28 | 21 | 17 | 10 | 6 |
| --- | --- | --- | --- | --- | --- | --- | --- | --- | --- |
| RGMI St. Petersburg | 68 | 25 | 11 | 8 | 6 | 2 | 0 | 0 | 0 |
